# Survival of the fewest: Microbial dormancy and maintenance in marine sediments through deep time

**DOI:** 10.1111/gbi.12313

**Published:** 2018-09-24

**Authors:** James A. Bradley, Jan P. Amend, Douglas E. LaRowe

**Affiliations:** ^1^ Department of Earth Sciences University of Southern California Los Angeles California; ^2^ Department of Biological Sciences University of Southern California Los Angeles California

**Keywords:** bioenergetics, dormancy, life in extreme environments, low energy, maintenance, numerical modelling

## Abstract

Microorganisms buried in marine sediments are known to endure starvation over geologic timescales. However, the mechanisms of how these microorganisms cope with prolonged energy limitation is unknown and therefore yet to be captured in a quantitative framework. Here, we present a novel mathematical model that considers (a) the physiological transitions between the active and dormant states of microorganisms, (b) the varying requirement for maintenance power between these phases, and (c) flexibility in the provenance (i.e., source) of energy from exogenous and endogenous catabolism. The model is applied to sediments underlying the oligotrophic South Pacific Gyre where microorganisms endure ultra‐low fluxes of energy for tens of millions of years. Good fits between model simulations and measurements of cellular carbon and organic carbon concentrations are obtained and are interpreted as follows: (a) the unfavourable microbial habitat in South Pacific Gyre sediments triggers rapid mortality and a transition to dormancy; (b) there is minimal biomass growth, and organic carbon consumption is dominated by catabolism to support maintenance activities rather than new biomass synthesis; (c) the amount of organic carbon that microorganisms consume for maintenance activities is equivalent to approximately 2% of their carbon biomass per year; and (d) microorganisms must rely solely on exogenous rather than endogenous catabolism to persist in South Pacific Gyre sediments over long timescales. This leads us to the conclusion that under oligotrophic conditions, the fitness of an organism is determined by its ability to simply stay alive, rather than to grow. This modelling framework is designed to be flexible for application to other sites and habitats, and thus serves as a new quantitative tool for determining the habitability of and an ultimate limit for life in any environment.

## 1 INTRODUCTION

Microorganisms in marine sediments are characterized by extreme energy limitation and slow metabolisms (Hoehler & Jørgensen, 2013; Lever et al., 2015). Marine sediments can thus be used to better understand constraints on the origin, proliferation and long‐term survival of life on Earth, while also serving as an analogue for extra‐terrestrial environments. Dormancy, a reversible state of low metabolic activity, is one strategy utilized by microorganisms to cope with perpetual energy limitation. Dormancy can contribute towards maintaining diversity in unpredictable and sub‐optimal habitats (Lennon & Jones, 2011), and is thought to be widespread among the subsurface biosphere (Jørgensen & Marshall, 2016). Once dormancy is initiated by starvation or resource limitation (Lennon & Jones, 2011), a cell's metabolism is reserved mostly to essential functions such as biomolecular repair and replacement, rather than to support growth (Kempes et al., 2017; Orcutt et al., 2013; Tijhuis, Van Loosdrecht, & Heijnen, 1993). “Maintenance” energy refers to the sum of the energetic costs of the activities that do not produce growth, but that are required to sustain life. These costs are poorly quantified in many environments (Hoehler & Jørgensen, 2013). Nevertheless, since most microorganisms in marine sediments appear to be merely surviving rather than growing, maintenance is thought to comprise a substantial component of the total power (the rate of energy utilization) that is consumed in these habitats (Bradley, Amend, & LaRowe, 2018a; LaRowe & Amend, 2015b; Orcutt et al., 2013).

The power that is available to microorganisms in an environment is dependent on the Gibbs energy harvested from the catalysis of redox reactions and the rate at which those reactions proceed. Catabolic reactions can generally be classified as endogenous, where cellular biomass is utilized as a reactant to serve as a source of energy (Dawes & Ribbons, 1962; Herbert, 1958), or exogenous, where the energy is supplied by the consumption of external substrates (Morita, 1988; Pirt, 1965). The operation of one strategy or the other by microorganisms is thought to be determined by environmental and thermodynamic factors (Wang & Post, 2012), and is enormously important in rates of carbon cycling and associated reactions in marine sediments (Arndt et al., 2013).

Presently, the extent of dormancy in the deep biosphere, as well as the role of endogenous and exogenous catabolism to support cellular maintenance, is virtually unknown. Numerous challenges to investigating life in the deep biosphere, including accessibility, measurement of extremely low biomass and rates of energy processing, and designing laboratory incubations whose conditions resemble the natural environment, have hindered progress in understanding these factors. Alternatively, mathematical models, which have long been used to describe microbial processes (reviewed in Bradley et al. 2018a; Bradley, Arndt, et al. 2016), have proven useful in providing a mechanistic framework with which to interpret observations (e.g., Dale, Bruchert, Alperin, and Regnier 2009). However, dormancy and maintenance processes in marine sediments have not been modelled, and there are presently no suitable numerical models that capture the details necessary to simulate these processes pertaining to sediments or the deep biosphere in a single framework (Bradley et al., 2018a).

Here, we present a new model “MicroLow 1.0” (Microbial Ecophysiology in Low Energy Environments 1.0) that includes novel mathematical constructs for microbial growth, yield, maintenance and physiological state, including a single active and multiple dormant phases. This model was designed with the marine sediment biosphere in mind, but it is also transferable to other transitory or low‐energy environments. The microbial model presented and implemented here is novel not only in its components and the processes that it simulates, but also in the multi‐million‐year timescales over which it is implemented. After describing the modelling framework, we simulate the biogeochemical dynamics of a deeply buried microbial community in oxic sediments underlying the South Pacific Gyre (SPG) and their role in particulate organic carbon (POC) degradation. The newly developed model is used to provide insight into the ecophysiology of these microorganisms and to suggest the means by which they may persist over extraordinarily long timescales, enduring prolonged energy limitation for upwards of millions of years.

## 2 METHODS

### 2.1 Microbial model

**Figure 1:**
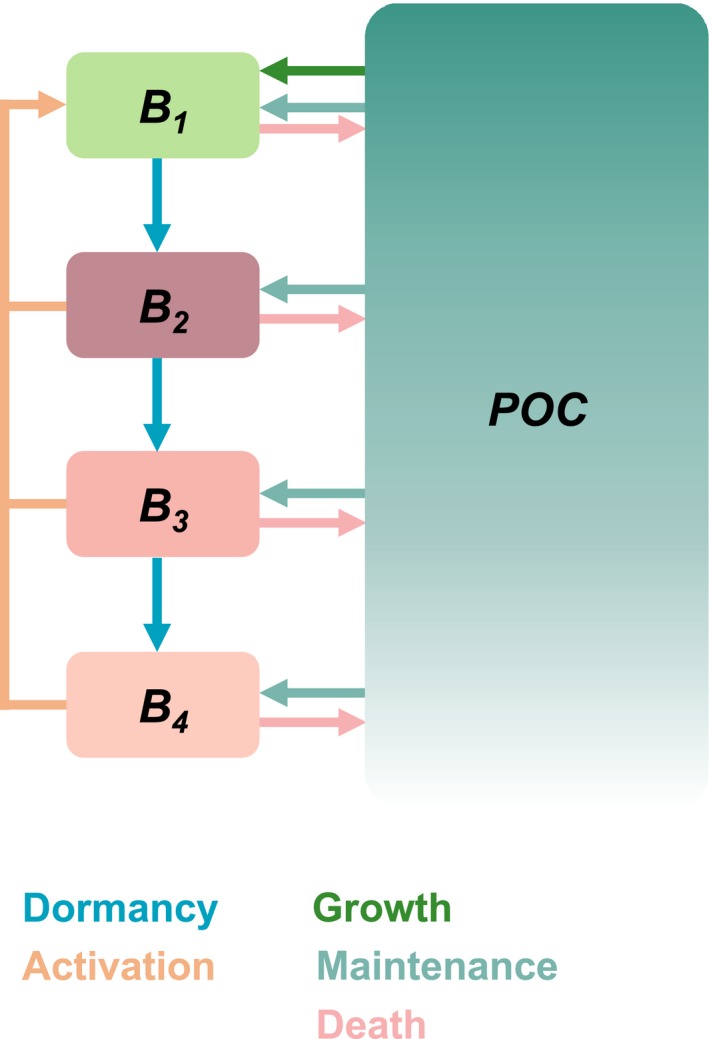
A conceptual model showing state variable components (boxes) and transfers among them (arrows). The state variables *B*_1_–*B*_4_ refer to pools of biomass that are distinguished by their physiological state. The state variable POC refers to particulate organic carbon. The arrows are colour‐coded according to the processes that increase or decrease the amount of carbon in each reservoir [Colour figure can be viewed at wileyonlinelibrary.com]

The microbial model implemented in this study divides microbial biomass, *B*, into four pools (*B*_1–4_), which are distinguished by their state of activity, and a single pool of organic matter representing POC (Table 1). A system of coupled ordinary differential equations (Table 2) describes the transfers and transformations of these pools due to growth, maintenance, activation, deactivation and death, according to the conceptual diagram shown in Figure 1. The state variables are listed in Table 1.

**Table 1:** State variables and initial values

State Variable	Description	Initial value (μg C/cm^3^)
*B*_1_	Active biomass: the only microorganisms capable of growth (i.e., cellular division).	0.040
*B*_2_	Biomass: first stage of dormancy.	0
*B*_3_	Biomass: second stage of dormancy.	0
*B*_4_	Biomass: third stage of dormancy.	0
POC	Particulate organic carbon.	781.26

**Table 2:** Model formulation

Fundamental balance equations	
Rate of change of *B*_1_	$\frac{\partial B_{1}}{\partial t}=V_{B1}-D_{B1}-\xi_{B1}-M_{En,B1}+\epsilon_{B2}+\epsilon_{B3}+\epsilon_{B4}$
Rate of change of *B*_2_	$\frac{\partial B_{2}}{\partial t}=-D_{B2}-\xi_{B2}+\xi_{B1}-\epsilon_{B2}-M_{En,B2}$
Rate of change of *B*_3_	$\frac{\partial B_{3}}{\partial t}=-D_{B3}-\xi_{B3}+\xi_{B2}-\epsilon_{B3}-M_{Ex,B3}$
Rate of change of *B*_4_	$\frac{\partial B_{4}}{\partial t}=-D_{B4}+\xi_{B3}-\epsilon_{B4}-M_{Ex,B4}$
Rate of change of POC	$\frac{\partial \text{POC}}{\partial t}=-\left(V_{B1}\cdot \frac{1}{Y_{G}}\right)-\sum M_{Ex,Bn}+\sum D_{\mathrm{Bn}}$
Microbial growth and death	
Growth of *B*_1_	$V_{B1}=B_{1}\cdot v_{\max}\cdot \left(\frac{\text{POC}}{K_{v}+\text{POC}}\right)$
Death of biomass *B*_*n*_	*D*_*Bn*_ = *α*_*Bn*_ · *B*_*n*_
Total death	∑ *D*_*Bn*_ = *D*_*B*1_ + *D*_*B*2_ + *D*_*B*3_ + *D*_*B*4_
Deactivation and activation	
Deactivation of biomass (*B*_*n*_)	$\xi_{\mathrm{Bn}}=\left(1-\theta_{S}\right)\cdot R_{S,D}\cdot B_{n}$
Activation of biomass (*B*_*n*_)	ϵ_*Bn*_ = *θ*_*S*_ · *R*_*S*,*A*_ · *B*_*n*_
Function to determine direction of change of state	$\theta_{S}=\frac{1}{e^{\left(\frac{-\mathrm{POC}+K_{S}}{st_{S}+K_{S}}\right)}+1}$
Maintenance	
Endogenous catabolism (*B*_*n*_)	$M_{En,Bn}=B_{n}\cdot m_{\mathrm{Bn}}\cdot \left(1-\theta_{M}\right)$
Exogenous catabolism (*B*_*n*_)	*M*_*Ex*,*Bn*_ = *B*_*n*_ · *m*_*Bn*_ · *θ*_*M*_
Total exogenous catabolism	∑ *M*_*Ex*_ = *M*_*Ex*,*B*1_ + *M*_*Ex*,*B*2_ + *M*_*Ex*,*B*3_ + *M*_*Ex*,*B*4_
Function to determine provenance of maintenance energy	$\theta_{M}=\frac{1}{e^{\left(\frac{-\mathrm{POC}+K_{M}}{st_{M}+K_{M}}\right)}+1}$

#### 2.1.1 Microbial dynamics

**Figure 2:**
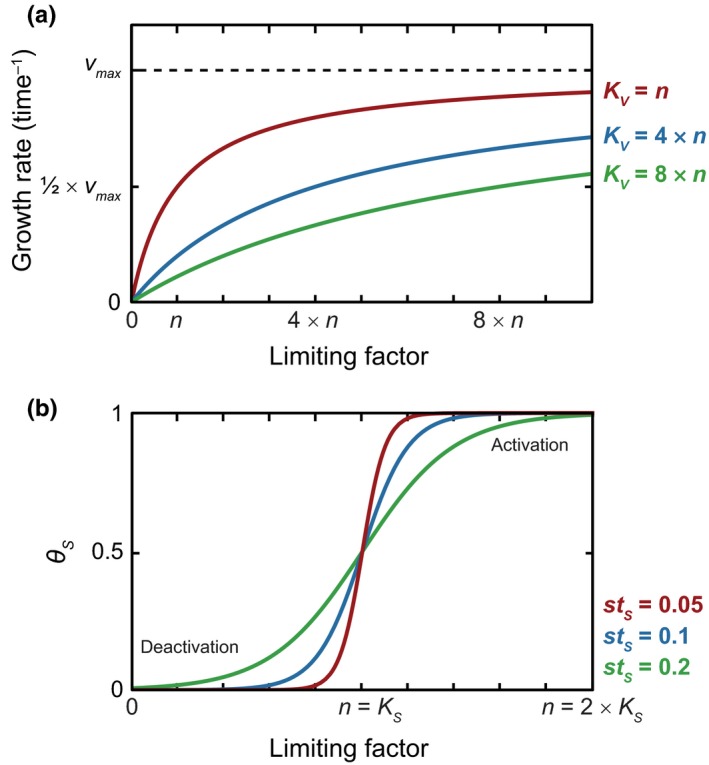
Illustration of how different values of model parameters influence how (a) growth rate depends on a limiting factor of concentration, *n*, for variable values of the half‐saturation constant, *K*_*V*_, according to Michaelis–Menten kinetics; and (b) values of *st*_*S*_ in the *θ*_*S*_ function determine the fraction of cells undergoing activation (*θ*_*S*_ >0.5) and deactivation (*θ*_*S*_ <0.5), as a function of the concentration of a limiting factor (*n*) and a threshold concentration (*K*_*S*_) [Colour figure can be viewed at wileyonlinelibrary.com]

We consider a single pool of active biomass (*B*_1_) and three pools of dormant biomass, from recently active (*B*_2_) to long since dormant (*B*_4_). It is intended that this framework represents a gradient (or depth) of dormancy across the microbial community (Lennon & Jones, 2011; Locey, 2010; Stolpovsky, Martinez‐Lavanchy, Heipieper, Van Cappellen, & Thullner, 2011). Note that we distinguish discreet pools for sake of a numerical description of this gradient and do not suggest the existence of three distinct dormancy states in natural settings. Active biomass is the only pool that is capable of growth (i.e., cellular division). It is assumed that aerobic heterotrophy is the dominant metabolism in oxic SPG sediments (D'Hondt et al., 2015), and thus, the growth rate of *B*_1_ is dependent on POC concentration, via Michaelis–Menten kinetics (Figure 2a) (Michaelis & Menten, 1913). Dormant cells are viable but must undergo activation before they are capable of growth. Dormancy requires that an organism (a) is not growing or dividing (i.e., has a reproductive rate equal to zero) and (b) has a lower metabolic demand than when it is active (Lennon & Jones, 2011; Stolpovsky et al., 2011). Furthermore, a dormant microorganism may better endure inhospitable conditions and thus have a lower mortality rate than its active counterpart (Johnson et al., 2007; Lennon & Jones, 2011; Price & Sowers, 2004). Organisms transition to a “deeper” state of dormancy the longer they have been dormant, decreasing their metabolic and mortality rates (Lennon & Jones, 2011; Stolpovsky et al., 2011). Stolpovsky et al. (2011) use a continuous function “S” to describe “depth of dormancy,” as a means to modify the mortality rate of dormant microorganisms based on the duration of unfavourable conditions. This necessitates the use of multiple additional parameters. Our approach, which is based instead on transitions between discreet pools, eliminates the need for such a function and requires fewer parameters. Biomass from all pools can die, utilize POC and biomass to fulfil maintenance power requirements, and transition to active and dormant phases.

The overall rate of change in active biomass (*B*_1_) is given by:

(1) $$\frac{\partial B_{1}}{\partial t}=V_{B1}-D_{B1}-\xi_{B1}-M_{En,B1}+\epsilon_{B2}+\epsilon_{B3}+\epsilon_{B4}$$

where *t* is time, *V*_*B*1_ represents the rate of new biomass growth, *D*_*B*1_ denotes the death rate of *B*_1_, *ξ*_*B*1_ corresponds to the deactivation rate of *B*_1_ into *B*_2_, *M*_*En,B*1_ represents the consumption of biomass due to endogenous catabolism of *B*_1_, and *ϵ*_*Bn*_ is the rate of activation of *B*_*n*_ into *B*_1_.

The overall change in all other biomass pools (*B*_*n*_) is given by:

(2) $$\frac{\partial B_{n}}{\partial t}=-D_{\mathrm{Bn}}-\xi_{\mathrm{Bn}}+\xi_{B\left(n-1\right)}-\epsilon_{\mathrm{Bn}}-M_{En,Bn}$$

where *D*_*Bn*_ represents the death rate of *B*_*n*_, *ξ*_*Bn*_ corresponds to the rate deactivation of *B*_*n*_ to *B*_(*n+*1)_, ξ_*B*(*n*−1)_ corresponds to the rate of deactivation of *B*_(*n−*1)_ to *B*_*n*_, *ϵ*_*Bn*_ is the activation of *B*_*n*_ to *B*_1_ *,* and *M*_*En,Bn*_ represents the consumption of biomass due to endogenous catabolism of *B*_*n*_.

Biomass growth is given by:

(3) $$V_{B1}=B_{1}\cdot v_{\max}\cdot \frac{\text{POC}}{K_{v}+\text{POC}}$$

where *B*_1_ is the concentration of active biomass in units of μg C/cm^3^ sediment, *v*_max_ is the maximum growth rate of active biomass, POC is the concentration of particulate organic carbon, and *K*_*v*_ is the half‐saturation constant for microbial growth according to standard Michaelis–Menten kinetics (Michaelis & Menten, 1913) (see Figure 2).

The microbial death rate is given by:

(4) $$D_{\mathrm{Bn}}=\alpha_{\mathrm{Bn}}\cdot B_{n}$$

where *α*_*Bn*_ is the mortality rate of biomass *B*_*n*_.

#### 2.1.2 Activation and deactivation

The model is based on the principle that microorganisms in marine sediments can take on various physiological states, from active and growing (*B*_1_) to dormant (*B*_2_ *–B*_4_) (Stolpovsky et al., 2011). The deactivation and reactivation of biomass (i.e., the transitioning of states between *B*_1_ and *B*_4_) depends on the potential supply of catabolic energy to the cells in relation to a threshold concentration of a limiting factor (*K*_*S*_), which is, in this case, POC concentration.

The deactivation (*ξ*) of biomass *B*_*n*_ to *B*_(*n*+1)_ is given by:

(5) $$\xi_{\mathrm{Bn}}=\left(1-\theta_{S}\right)\cdot R_{S,D}\cdot B_{n}$$

where *R*_*S,D*_ is the specific rate of deactivation. Similarly, the activation (*ϵ*) of biomass *B*_*n*_ to *B*_1_ is given by:

(6) $$\epsilon_{\mathrm{Bn}}=\theta_{S}\cdot R_{S,A}\cdot B_{n}$$

where *R*_*S,A*_ is the specific rate of activation.

*θ*_*S*_ is a function that accounts for the direction and rate of state change depending on POC as a limiting resource. It is based on the principle that if conditions in the environment are better than a certain threshold, there will be net activation of biomass and vice versa. The function, from Stolpovsky et al. (2011), is adapted from Fermi–Dirac statistics:

(7) $$\theta_{S}=\frac{1}{e^{\left(\frac{-\mathrm{POC}+K_{S}}{st_{S}+K_{S}}\right)}+1}$$

where *K*_*S*_ is a threshold POC concentration for net activation–deactivation, and *st*_*S*_ is a non‐dimensional parameter controlling the steepness of the sigmoidal function as shown in Figure 2b. There is a net deactivation of biomass (i.e., *B*_*n*_ to *B*_(*n*+1)_) under unfavourable conditions when the concentration of POC falls below *K*_*S*_ and thus *θ*_*S*_ < 0.5. Similarly, there is a net activation of biomass (i.e., *B*_*n*_ to *B*_1_) when the concentration of POC rises above *K*_*S*_ and thus *θ*_*S*_ > 0.5. Unlike deactivation, where cells must first transition through the first stages of dormancy to enter into a deeper state of dormancy (Lennon & Jones, 2011; Stolpovsky et al., 2011), dormant microorganisms from any state transition directly to the active state (*B*_1_) upon the onset of favourable conditions at a rate determined by *θ*_*S*_ and *R*_*S,A*_ (Morono et al., 2011; Takano et al., 2010).

#### 2.1.3 Maintenance

Both active and dormant cells are capable of endogenous (i.e., biomass derived) and exogenous (i.e., external substrate derived) catabolism (Bradley et al., 2018a; Gonzalez‐Pastor, Hobbs, & Losick, 2003; Kadouri, Jurkevitch, Okon, & Castro‐Sowinski, 2005; Rao, Alonso, Rand, Dick, & Pethe, 2008). The rate of endogenous catabolism, *M*_*En*_ *,*_*Bn*_, is described by:

(8) $$M_{En,Bn}=B_{n}\cdot m_{\mathrm{Bn}}\cdot \left(1-\theta_{M}\right)$$

where *m*_*Bn*_ is the specific maintenance power requirement of *B*_*n*_. Maintenance power requirements (*m*_*Bn*_) are described as a proportional carbon cost per unit of biomass per thousand years (i.e., similar to growth (*v*_max_) and mortality (*α*_*Bn*_) rates). Exogenous catabolism (for maintenance) is described by:

(9) $$M_{Ex,Bn}=B_{n}\cdot m_{\mathrm{Bn}}\cdot \theta_{M}$$

Similar to Equation (7), *θ*_*M*_ represents a sigmoidal function that allows for flexible exogenous and endogenous catabolism, depending on the concentration of POC:

(10) $$\theta_{M}=\frac{1}{e^{\left(\frac{-\mathrm{POC}+K_{M}}{st_{M}+K_{M}}\right)}+1}$$

where *K*_*M*_ is a threshold POC concentration determining the provenance (i.e., source) of maintenance power from biomass (*θ*_*M*_ **< 0.5) or substrate (*θ*_*M*_ > 0.5), and *st*_*M*_ is a non‐dimensional parameter controlling the steepness of the sigmoidal function as shown in Figure 2b. Under non‐critical conditions (i.e., POC > *K*_*M*_), *θ*_*M*_ approaches 1, and the majority of maintenance power is derived from exogenous catabolism. Under critical conditions where POC nears or is lower than *K*_*M*_, *θ*_*M*_ approaches 0, and endogenous catabolism is the dominant source of energy for maintenance activities.

#### 2.1.4 Particulate organic carbon dynamics

The rate of POC degradation is given by:

(11) $$\frac{\partial \mathrm{POC}}{\partial t}=-\left(V_{B1}\cdot \frac{1}{Y_{G}}\right)-\sum M_{Ex,Bn}+\sum D_{\mathrm{Bn}}$$

where *Y*_*G*_ represents the true growth yield (i.e., the yield in absence of maintenance (Bradley et al., 2018a; Heijnen & Van Dijken, 1992; Pirt, 1965; Van Bodegom, 2007)), *M*_*Ex,Bn*_ represents the rate of exogenous consumption of substrate for maintenance by *B*_*n*_, and *D*_*Bn*_ represents the rate at which dead cells contribute POC from *B*_*n*_ (i.e., necromass).

### 2.2 Model application

We selected sediments underlying the SPG for a detailed case study site to model microbial ecophysiology over multi‐million‐year timescales (D'Hondt et al., 2009, 2015). Ocean primary productivity in the SPG is so low that organic carbon concentrations in underlying sediments are three orders of magnitude lower (~0.01%) than those underlying productive areas of the open ocean and continental margins (2%–10%) (D'Hondt et al., 2015; Lomstein, Langerhuus, D'Hondt, Jørgensen, & Spivack, 2012; Parkes et al., 1993). Sediments that have been isolated for up to 75 million years sustain thousands of microorganisms per cm^3^, despite these ultra‐low concentrations of organic matter. It is thought that the microorganisms inhabiting these sediments persist in some of the lowest energy states on Earth (LaRowe & Amend, 2015b). For the purpose of this study, we have selected measurements of cell abundance and POC concentration from drill hole U1370 to test model output (IODP Expedition 329, D'Hondt, Inagaki, and Alvarez Zarikian (2011)). We focus on this site in particular due to its suitability for exploring microbial habitats and metabolisms in oligotrophic and low‐energy sediments (D'Hondt et al., 2015). Furthermore, this site has previously been used in comprehensive analyses of microbial carbon cycling, bioenergetics, and power availability and utilization in the marine subsurface (Bradley, Amend, & LaRowe, 2018b; D'Hondt et al., 2015; LaRowe & Amend, 2015a,b).

### 2.3 Initial values and test data

Cell abundance and POC concentrations are taken from published analyses of extracted drill cores (D'Hondt et al., 2011, 2015). To convert sediment depth (*z*) to sediment age (age*(z)*), we use the following equation (Berner, 1980):

(12) $$\text{age}\left(z\right)=\frac{z+\frac{\Phi_{0}}{c_{0}}\left(e^{-c_{0}\cdot z}-1\right)}{\omega_{0}\left(1-\Phi_{0}\right)}$$

where Φ_0_ represents the porosity at the sediment–water interface (SWI), *c*_0_ corresponds to the compaction length scale, and *ω*_0_ refers to the sedimentation rate, which is assumed to be constant. The porosity at depth *z*,* Φz*, is calculated by (Athy, 1930):

(13) $$\Phi_{z}=\Phi_{0}\cdot e^{-c_{0}\cdot z}$$

We use a SWI porosity (Φ_0_) equal to 0.87, a uniform sedimentation rate (*ω*_0_ = 10^−6^ m/year) and grain density (2.3 g/cm^3^), and a compaction length scale (*c*_0_) of 0.00085 per m (Burwicz, Rüpke, & Wallmann, 2011; D'Hondt et al., 2011).

We assume a cell mass of 14 fg C/cell, consistent with recent estimates for microbial cells in marine sediments (Braun et al., 2016; Kallmeyer, Pockalny, Adhikari, Smith, & D’Hondt, 2012) and a bioenergetic analysis of SPG sediments (Bradley et al., 2018b). POC provided in weight % is converted into μg C_org_ per cm^3^ for every depth accounting for changes in porosity using the physical model described above. We used regression models from a previous study (Bradley et al., 2018b) to provide initial values (i.e., *t*_0_) for microbial abundance and POC (*R* ^2^ > 0.97) (Table 1).

### 2.4 Implementation and numerical solution

The mathematical expressions described above and in Table 2 are implemented in the open‐source computing environment and programming language R, which is freely available (http://www.r-project.org/). Model code is available for free online via the Supporting Information Appendix S1.

The model is run with nominal parameters for a period representing 75 million years. Parameter values are constrained from results published in other studies (Table 3). A sensitivity study of 16 model parameters is carried out to assess the stability of model output and the dependency of output on individual parameters. Every parameter is sequentially adjusted by +5% of the nominal value and tested, and then returned to its nominal value before testing the next one. Results of the sensitivity tests are compared to the baseline simulation in which the model parameters are equal to those shown in Table 3.

**Table 3:** Parameters. Note that the units of time‐dependent parameters are provided per thousand years

Parameter	Description	Units	Nominal value	Reference
*v*_max_	Maximum growth rate of *B*_1_	Per thousand years	0.173	Lomstein et al. (2012)
*K*_*v*_	Half‐saturation constant for growth, based on POC concentration	μg C/cm^3^	40,000	This study
*Y*_*G*_	True growth yield (i.e., in the absence of maintenance)	Unitless	0.2	Jørgensen and Marshall (2016); Whitman et al. (1998)
*m*_*B*1_	Maintenance demand of *B*_1_	Per thousand years	23	Bradley et al. (2018b)
*m*_*B*2_	Maintenance demand of *B*_2_	Per thousand years	19	Bradley et al. (2018b)
*m*_*B*3_	Maintenance demand of *B*_3_	Per thousand years	15	Bradley et al. (2018b)
*m*_*B*4_	Maintenance demand of *B*_4_	Per thousand years	11	LaRowe and Amend (2015b)
*α*_*B*1_	Mortality rate of *B*_1_	Per thousand years	0.00350	Bradley et al. (2018b)
*α*_*B*2_	Mortality rate of *B*_2_	Per thousand years	0.00082	Bradley et al. (2018b)
*α*_*B*3_	Mortality rate of *B*_3_	Per thousand years	0.00011	Bradley et al. (2018b)
*α*_*B*4_	Mortality rate of *B*_4_	Per thousand years	0.000014	Bradley et al. (2018b)
*st*_*S*_	Steepness of state‐change dependency	Unitless	0.1	Stolpovsky et al. (2011)
*K*_*S*_	Threshold POC concentration for state change	μg C/cm^3^	10,000	This study
*R*_*S,D*_	Rate constant for deactivation	Per thousand years	0.0001	This study
*R*_*S,A*_	Rate constant for activation	Per thousand years	0.0001	This study
*st*_*M*_	Steepness of maintenance power dependency	Unitless	0.1	Stolpovsky et al. (2011)
*K*_*M*_	Threshold POC concentration for maintenance energy provenance	μg C/cm^3^	15	This study

## 3 RESULTS

### 3.1 Microbial and geochemical dynamics in SPG sediments

**Figure 3:**
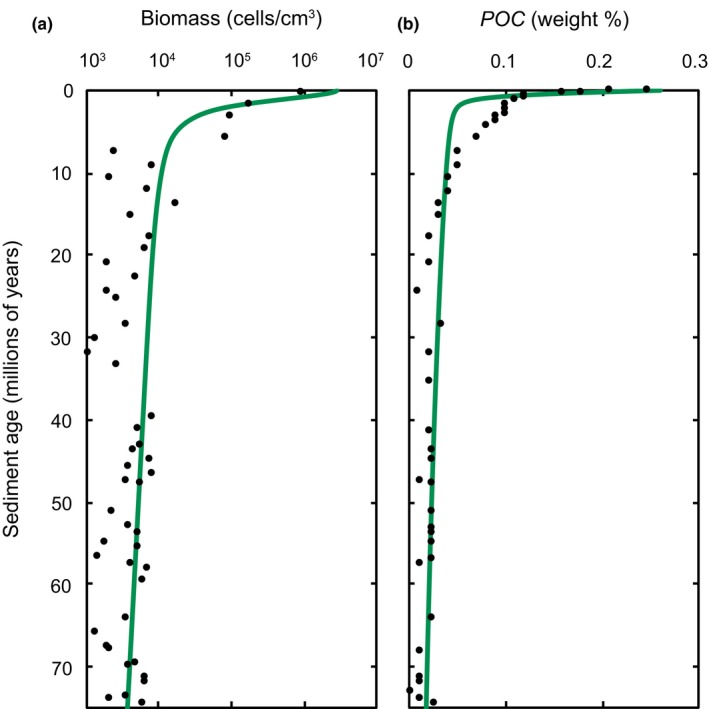
Observed (points, D'Hondt et al., 2011, 2015) and modelled (lines) (a) biomass and (b) POC concentrations in marine sediments at U1370 in the South Pacific Gyre [Colour figure can be viewed at wileyonlinelibrary.com]

Figure 3 summarizes the change in cell abundance and POC concentration in SPG sediments simulated over a period of 75 million years. Both simulation results and experimental data show that cell concentration is characterized by an initial phase of relatively rapid cell abundance decline from ~2.8 × 10^6^ cells/cm^3^ to ~6.0 × 10^5^ cells/cm^3^ over 1 million years (Figure 3a). After ~5 million years, the rate of cell abundance decline decreases. In extremely ancient sediments (~40 to 75 million years following burial), cell abundance declines extremely slowly, and the sediments support a relatively stable concentration of ~100 to 1,000 cells/cm^3^.

**Figure 4:**
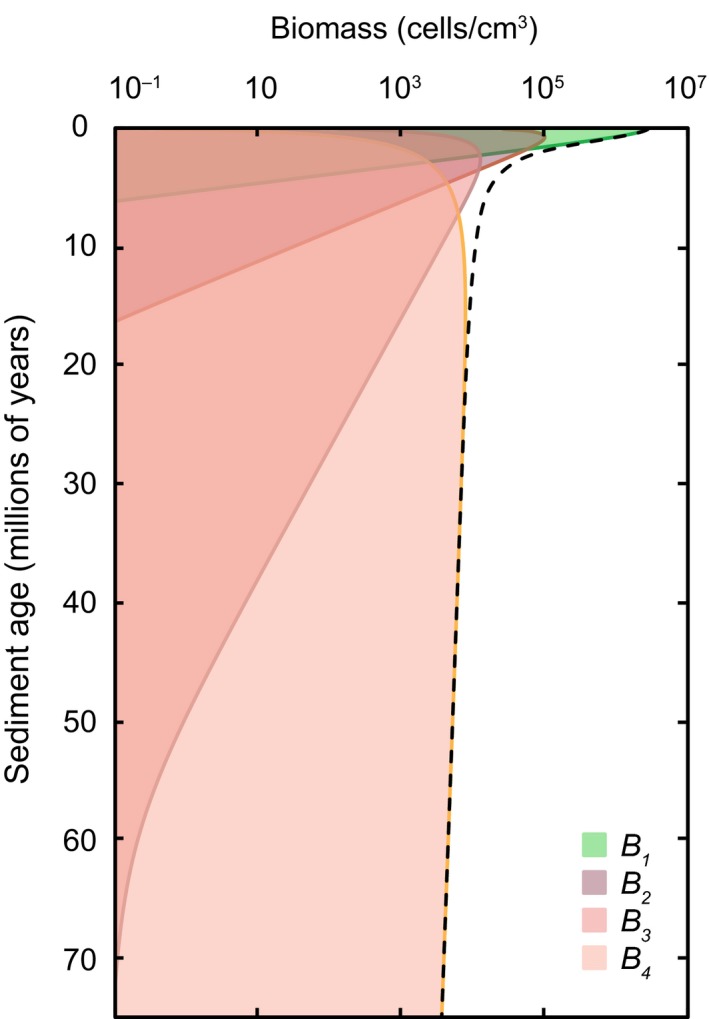
Simulated total biomass (dashed line) in SPG sediments and the physiological state of this biomass over 75 million years of burial, as shown by the proportion of biomass in each physiological group (shaded colours) [Colour figure can be viewed at wileyonlinelibrary.com]

The decline in cell abundance over 75 million years is characterized by a shift from active (*B*_1_) to dormant (*B*_2_) cells in the youngest sediments (<1 million years) (Figure 4). Dormant cells (*B*_2_) then transition to increasingly dormant states (*B*_3_, *B*_4_) over the tens of millions of years that follow.

There is a continual depletion in POC (Figure 3b) in the sediments underlying SPG that is reflected in both simulation results and experimental data. POC initially drops from ~0.26 to ~0.05 weight % during the first ~2 million years of burial. Following this initial period, further degradation of POC proceeds extremely slowly and a concentration of <0.04 weight % is preserved to the bottom of the sediment core, which is ~75 million years old.

**Figure 5:**
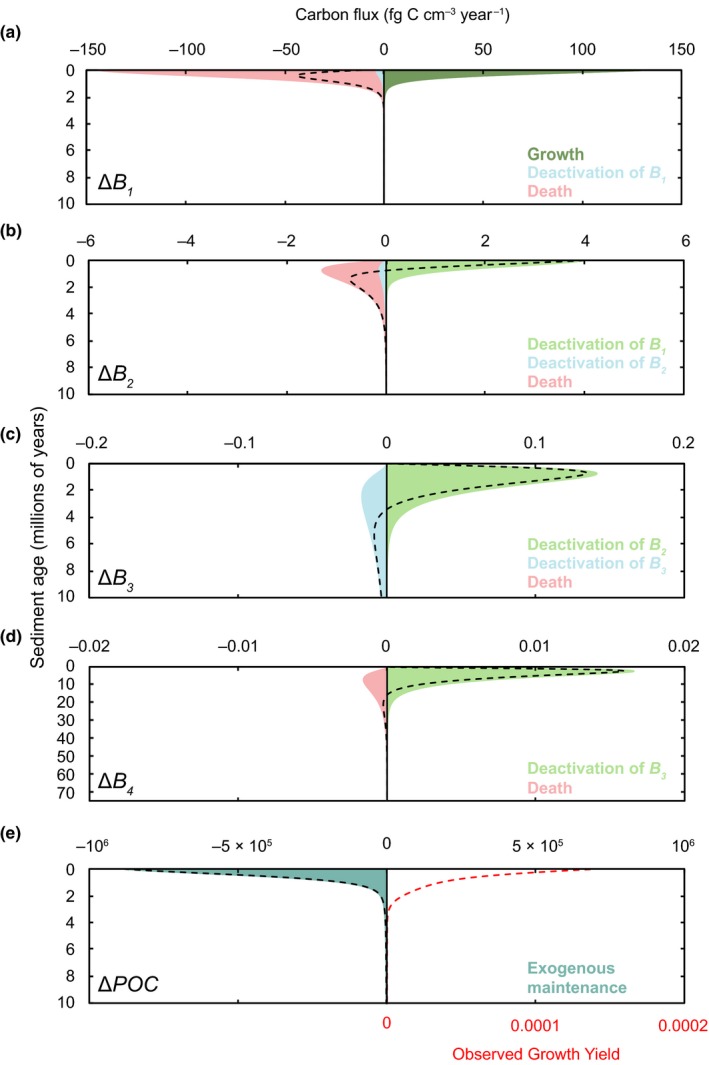
Flux of carbon in and out of (a–d) each biomass pool (*B*_1_ to *B*_4_) resulting from growth, maintenance, death and the deactivation of microbial groups into various stages of dormancy (shaded regions). Flux of carbon in the (e) POC pool results from exogenous maintenance (turquoise), growth and necromass contributions. Fluxes due to growth and necromass are extremely small in magnitude (<160 fg C cm^−3^ year^−1^) and thus are not visible on this scale. The net change in carbon is represented by the dashed black line (a–e). Increases in biomass are due to growth (dark green) and the deactivation of less dormant biomass pools (light green). The loss of biomass arises from transitions to a more dormant state (blue) and death (pink). There is no activation of biomass (e.g., transition from *B*_*n*_ to *B*_1_) or consumption of biomass for maintenance (endogenous catabolism). (e) Observed growth yield (red) [Colour figure can be viewed at wileyonlinelibrary.com]

The various microbiological processes that are responsible for the shifts in physiological state (Figure 4), as well as POC concentration (Figure 3), are quantified at each time step in the model. The magnitude of these processes at every depth and the net effect on each state variable are illustrated in Figure 5. For instance, it can be seen in Figure 5a that high rates of mortality (<140 fg C cm^−3^ year^−1^) for *B*_1_ and the transition of active cells into a dormant state (*B*_1_ to *B*_2_) (<4 fg C cm^−3^ year^−1^) outweigh the flux of new carbon into *B*_1_ from the growth of active cells (<130 fg C cm^−3^ year^−1^). Thus, there is a net depletion in *B*_1_, which is most prominent over the first 2 million years of burial. The deactivation of cells (i.e., transition from *B*_1_ to *B*_2_) contributes to an increase in the concentration of dormant cells (Figure 5b), and thus, the net change in *B*_2_ is initially positive. However, relatively high mortality of *B*_2_ and the transition of *B*_2_ to a deeper state of dormancy (*B*_3_) cause an overall net loss of *B*_2_ following 750,000 years of burial. As microorganisms transition to the deeper states of dormancy (*B*_3_, *B*_4_), the contribution of deactivating organisms generally outweighs the mortality of those organisms, and thus, the net change for these groups is positive, indicating that the more dormant microorganisms are mostly being preserved in older sediments. Microorganisms that are in the deepest state of dormancy (*B*_4_) have a low mortality rate and are generally preserved in the sediment longer than those that were more recently active. There is no activation of biomass (*ϵ*, i.e., *B*_*n*_ to *B*_1_) throughout the duration of the simulation—only a transition to a more dormant state.

Model results show that nearly all of the carbon processed by microorganisms is dedicated to maintenance activities (<886,000 fg C degraded cm^−3^ year^−1^) rather than growth (<140 fg C degraded cm^−3^ year^−1^) (Figure 5e). Endogenous catabolism is negligible, at a magnitude that is too small to appear in Figure 5. Additionally, the autochthonous (i.e., formed in situ) necromass‐derived POC is negligible (<156 fg C cm^−3^ year^−1^).

### 3.2 Endogenous catabolism

**Figure 6:**
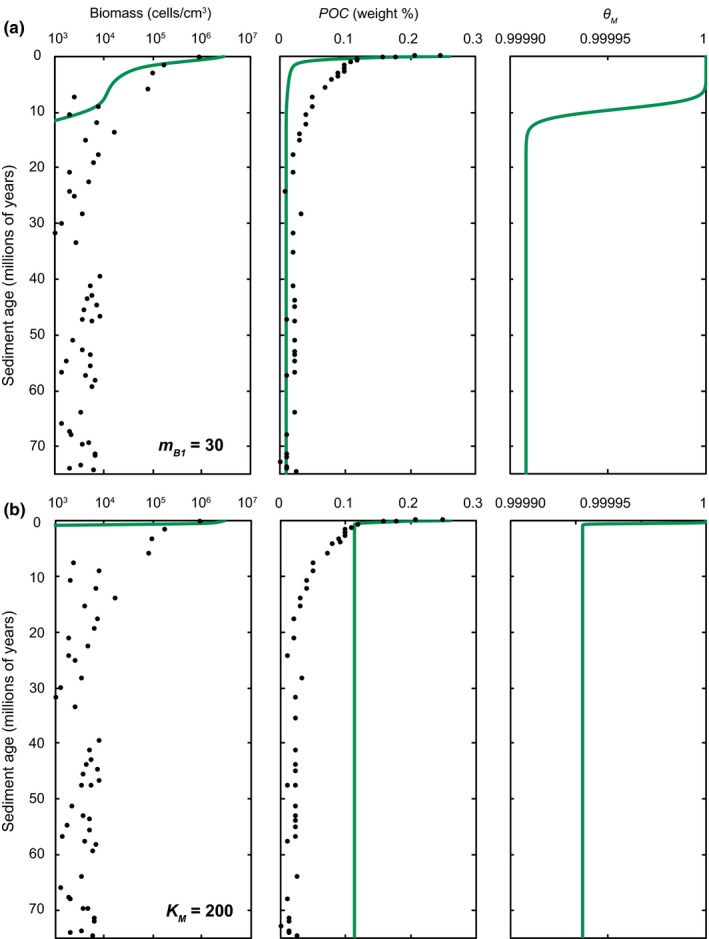
Biomass and POC concentrations for simulations in which (a) the maintenance requirement for the most active biomass fraction, *m*_*B*1_, is increased from 23 (per thousand years) to 30 (per thousand years), and (b) the critical threshold POC concentration for a preference of exogenous to endogenous catabolism (*K*_*M*_) is raised from 15 to 200 μg C/cm^3^. All other parameters remain at nominal values. The resulting values of *θ*_*M*_, the function for the source of the maintenance power, are also shown [Colour figure can be viewed at wileyonlinelibrary.com]

Two additional simulations are performed in which parameters were manipulated to simulate an environment where microorganisms are more likely to utilize their own biomass as a source of energy in SPG sediments (i.e., a preference of endogenous catabolism over exogenous catabolism). In the first simulation, illustrated in Figure 6a, the maintenance power demand of *B*_1_ (*m*_*B*1_) is increased from 23 (per thousand years) to 30 (per thousand years). The immediate effect of increasing the microorganisms’ demand for maintenance energy is an increase in the rate of POC consumption. POC is rapidly exhausted, approaching the critical threshold level for endogenous catabolism (*K*_*M*_), and the *θ*_*M*_ function controlling the preference of endogenous vs exogenous catabolism (*θ*_*M*_) is lowered (<1) (Figure 6a). The moderate decrease in *θ*_*M*_ that occurs between 5 and 12 million years following burial is sufficient for microorganisms to partially utilize biomass for maintenance activities. This effect causes a rapid depletion of biomass due to endogenous catabolism, essentially exhausting all biomass carbon in sediments >10 million years old.

In the second simulation, shown in Figure 6b, the critical threshold POC concentration for endogenous versus exogenous catabolism (*K*_*M*_) is raised from 15 μg C/cm^3^ to 200 μg C/cm^3^. Depletion in biomass and consumption of POC occurs in a similar fashion to the baseline simulation for ~400,000 years, after which POC ≈ *K*_*M*_, thus lowering the *θ*_*M*_ function regulating the amount of maintenance power coming from exogenous versus endogenous sources (*θ*_*M*_ < 1). Under these conditions, microorganisms obtain a proportion of the energy that supports their maintenance activities from their own biomass instead of residual sediment POC. Values of *θ*_*M*_ slightly lower than 1 cause a rapid depletion of biomass, effectively eliminating all life in fewer than one million years.

### 3.3 Sensitivity of model parameters

**Figure 7:**
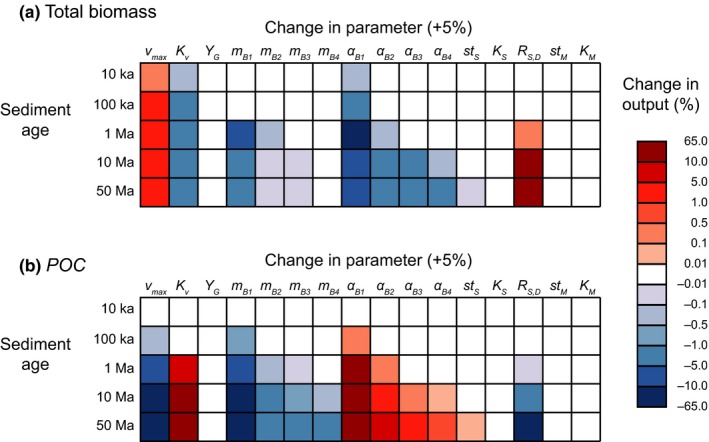
The sensitivity of model output, (a) total biomass and (b) POC concentration at various sediment ages at SPG, to 5% increases in nominal parameter values (while all other parameters held at their nominal values). See text and Table 3 for nominal values of the model parameters and what they represent [Colour figure can be viewed at wileyonlinelibrary.com]

The sensitivity of (a) total biomass and (b) POC concentrations to changes in individual model parameters across a range of sediment ages (10,000 to 50 million years) are illustrated in Figure 7. Each box in this figure corresponds to how much model results were altered by increasing each of the indicated parameters one at a time by 5%. The shading of each box indicates the sign and magnitude of percentage change in model output compared to the baseline simulation. In general, biomass concentrations are more sensitive to parameter variation in older sediments than in younger sediments, indicated by the darker shading for 50 million and 10 million‐year‐old sediments compared to younger (10,000, 100,000 and one million year old) sediments.

The concentration of biomass is most sensitive to variation in mortality rates (*α*_*B*1–4_, up to −10.1% change) and the rate of transition between physiological states (*R*_*S,D*_, up to 12.0%). Biomass concentration is also sensitive to variation in growth rate (*v*_*max*_, up to 4.6%), the half‐saturation constant for growth (*K*_*v*_, up to −4.6%) and cellular maintenance power demand (*m*_*B*1–4_, up to −5.0%).

The sensitivity of POC to parameter variation generally mirrors the sensitivity of biomass, but notably, the sign of the change in the model output is opposite for all parameters except maintenance power demand (*m*_*Bn*_). Hence, whereas an increase in *v*_max_ causes a considerable increase in biomass throughout all ages of the sediment, it has the opposite effect on POC—causing it to be consumed more rapidly, and thus, concentrations of POC are lower than the baseline simulation and the data. Similarly, increased mortality (*α*_*Bn*_) considerably depletes biomass concentrations relative to the baseline simulation, but more POC remains. Variation in the true growth yield (*Y*_*G*_), the steepness of the physiological state and maintenance response functions (*st*_*S*_ and *st*_*M*_, Equations (7) and (10)), and threshold constants for physiological state change and maintenance (*K*_*S*_ and *K*_*M*_) do not noticeably affect the simulated biomass and organic carbon concentrations over 75 million years of burial (indicated by faded colours or white).

## 4 DISCUSSION

### 4.1 Model results

Microorganisms are found to inhabit remote and oligotrophic marine sediments at very low abundances (~10^3^ to 10^4^ cells/cm^3^) for extraordinarily long timescales (> 20 million years) (D'Hondt et al., 2015). Just the fact that intact microbial cells are found in this ancient habitat has remarkable implications concerning the resilience of these organisms, yet almost nothing is known about the physiological mechanisms concerning their growth, death and activity. The new model presented and implemented here, which is the first model to explicitly represent microbial dormancy in the deep biosphere, is an attempt to reconcile these unknowns, using a quantitative and mechanistic approach. Thus, we provide a process‐based account of the mechanisms that might enable microorganisms to persevere in these settings and endure extreme energy limitation over prolonged timescales.

First, model results suggest that marine sediments at SPG constitute an unfavourable habitat for the growth and proliferation of microorganisms. This is based on our interpretation of biomass concentrations, which exhibit a sharp decline over several orders of magnitude during the initial ~5 million years following deposition. The most favourable setting (in terms of total catabolic power supply) in SPG sediment occurs near the SWI where the highest concentrations of POC and oxygen are (D'Hondt et al., 2011). Nevertheless, at this locale, mortality rates are highest and new biomass growth is insignificant (Figure 5). Thus, we infer that the vast majority of microorganisms that are entombed in sediments at SPG are poorly suited to this environment. This is supported by empirical measurements of cell abundance at SPG (Figure 3). Moreover, the rapid decline in biomass in recently deposited near‐surface sediment is a global phenomenon (Jørgensen & Marshall, 2016), suggesting that the low‐energy environment typical of sediments is not favourable to the organisms found inhabiting them.

Second, model results are suggestive that dormancy is prevalent in the SPG subsurface and that the vast majority of these microorganisms exist in a state of reduced metabolic activity. Microorganisms are initially prescribed to a metabolically “active” state (*B*_1_), that is they are capable of growth. However, in response to unfavourable environmental conditions, the vast majority of microorganisms either die or rapidly transition to a dormant (*B*_2–4_) state, with minimal new biomass growth. This response is reflected in laboratory experiments and in other models simulating microbial dormancy (Blagodatsky, Heinemeyer, & Richter, 2000; Blagodatsky & Richter, 1998; Blagodatsky, Yevdokimov, Larionova, & Richter, 1998; Konopka, 1999; Lennon & Jones, 2011; Stenström, Svensson, & Johansson, 2001; Stolpovsky et al., 2011). The near‐zero growth of deeply buried microorganisms, as well as the prevalence of dormancy among the community, leads to the premise that these cells are ancient “relic organisms,” comprised of a few rare members of the surface microbial community that survived burial. This interpretation is corroborated by evidence from comparative DNA amplicon, single‐cell and metagenomic sequencing in marine sediments from Aarhus Bay, Denmark, showing that deep sub‐seafloor sediments (>5,000 years old) are populated by a select few descendants and survivors of microbial communities from surface sediments (Starnawski et al., 2017).

In many habitats, microorganisms experiencing favourable changes in their environment may exit the dormant state and become active once again (Lennon & Jones, 2011; Morono et al., 2011; Takano et al., 2010; Trembath‐Reichert et al., 2017). However, despite incorporating a numerical description of this phenomenon in the model presented here, microorganisms transition only from an active state to a dormant/increasingly dormant state, and not vice versa. Consequently, based on model results, we suggest that there is no (or at most, minimal) activation of dormant cells that occurs in these sediments, since conditions only worsen with increased burial. It is the continual worsening of conditions over time and burial that has led to dormancy in the marine subsurface being likened to a “dead‐end strategy” (Jørgensen, 2012). The estimated generation times for the deep biosphere, up to several thousands of years (Braun et al., 2017; Trembath‐Reichert et al., 2017; Biddle et al., 2006; Jorgensen, D'Hondt, & Miller, 2006; Jørgensen, 2011; Lomstein et al., 2012; Whitman, Coleman, & Wiebe, 1998; Xie, Lipp, Wegener, Ferdelman, & Hinrichs, 2013), necessitate very low mutation rates, and therefore, selection is likely dominated by the presence of pre‐adapted sub‐seafloor taxa (Orsi, 2018; Petro, Starnawski, Schramm, & Kjeldsen, 2017). Further, whether these generation times reflect actual growth (i.e., new biomass generation from cellular division) or replacement (i.e., turnover of biomolecules without division, akin to maintenance activities) is not known. Accordingly, organisms in the deep biosphere may effectively be precluded from the evolutionary emergence of advantageous traits since this requires cellular growth and division. In accordance with previous findings (Lennon & Jones, 2011; Stolpovsky et al., 2011), our results suggest that cells that are able to successfully transition to dormancy may outcompete organisms exhibiting a “fast growth” strategy in ultra‐oligotrophic settings such as SPG, thereby enabling their persistence over extremely long timescales.

Third, model results lead us to believe that POC consumption is dominated by catabolism to support maintenance activities rather than new biomass synthesis (i.e., growth). The decline in cell abundance is accompanied by a decline in POC concentration, which, in both modelled and experimental data, is most rapid near the SWI (Figure 3). Modelling studies (D'Hondt et al., 2015; LaRowe & Amend, 2015b) and measurements (D'Hondt et al., 2009, 2011, 2015) suggest that microorganisms at SPG utilize this POC as an electron donor coupled to the reduction in oxygen as a primary source of energy. The main source of POC to microorganisms at SPG is allochthonous material that was deposited and buried along with the inoculum of microbial cells, rather than POC supplied autochthonously in the form of necromass (Figure 5). This is corroborated by a previous finding that despite relatively high mortality, the autochthonous recycling of microbial necromass is a negligible source of power at SPG (Bradley et al., 2018b). The rate of POC degradation, which is initially rapid, noticeably decreases between 2 and 10 million years. In both model predictions and measurements, a minor fraction of POC (0.01–0.1 weight %) is preserved over extended timescales (~75 million years). Observed growth yield at SPG declines from ~0.00015 in shallow sediment to effectively zero in sediments older than 4 million years (Figure 5e). These values are remarkably low compared to other environments such as other marine sediments (0.08–0.20 (Starnawski et al., 2017; Whitman et al., 1998)) and soils (0.06–0.84 (Bradley, Anesio, & Arndt, 2016; Blagodatsky et al., 1998)). Thus, we interpret a near no‐growth scenario in shallow SPG sediments, and a no‐growth scenario in sediments older than 4 million years (Bradley et al., 2018a). Our deduction of maintenance‐dominated catabolism is consistent with a number of substrate‐ or energy‐limited environments where cells do not grow despite being known to consume POC (Blagodatsky et al., 2000; del Giorgio & Cole, 1998). Our interpretation of these results also aligns with the findings of theoretical studies in which the maintenance activity of increasingly small and starving prokaryotes converges on total activity, since energy expenditure is dominated by biomolecule repair and replacement rather than new biomass synthesis (Kempes, Dutkiewicz, & Follows, 2012; Kempes et al., 2017; Maitra & Dill, 2015).

Furthermore, we believe that it is possible that the low rates of microbial activity prescribed to the model are in fact too high. There is generally good agreement between modelled POC concentration and measured POC from SPG sediment cores; however, a notable mismatch occurs between ~2 and 8 million years following burial, where the model predicts POC concentrations lower than those measured. It is likely, therefore, that simulated POC degradation rates exceed the actual POC degradation rates over this interval, and thus, by extension, simulated microbial maintenance activity is too high. This idea challenges our understanding of the minimum power requirement of microbial life under extreme energy limitation, which has been suggested to be as low as 10^−21^ J s^−1^ cell^−1^ (LaRowe & Amend, 2015b). Furthermore, it has been shown that the distribution of activity among a population of starving microorganisms may be highly negatively skewed (i.e., a small number of cells utilizing a large proportion of total energy) (Shoemaker & Lennon, 2017). This possibility is not accounted for in our estimation of power per cell and may have ecological consequences on the POC profile and the interpretation of results. By focusing on implementing various versions of eco‐physiological models for microorganisms in extremely energy‐limited settings, and also capturing the variability and distribution of activity within a community, we may improve the understanding of the minimum power that is required to support microbial life.

Finally, modelling results presented here clearly reveal that POC in SPG sediments plays a critical role in the supply of catabolic power for an organism to undertake maintenance activities, thus enabling its survival. We deduce that microorganisms must rely solely on exogenous catabolism rather than endogenous catabolism to be able to persist in SPG sediments over millions of years. In two additional simulations designed to test a strategy whereby microorganisms partially rely on endogenous catabolism (i.e., biomass used in place of POC as an electron donor in oxygenic respiration), the community rapidly declines to extinction (at ~1–12 million years). Thus, it is clear that microorganisms in SPG sediments are not utilizing biomass to supply maintenance power (endogenous catabolism), since these manipulation experiments do not reproduce empirical evidence of microbial life in sediments that are tens of millions of years old. Thus, we deduce that an exogenous catabolic strategy is favoured, or even essential, under highly energy‐limiting conditions to maintain a population of cells that persist for millions of years. This result corroborates previous suggestions that dormant microorganisms that deplete their internal reserves will die without replenishment of those resources (Lennon & Jones, 2011).

Microbial metabolisms in oxic sediments are dominated by the heterotrophic degradation of organic matter with oxygen, with communities comprised predominantly of aerobic and facultative anaerobic heterotrophs affiliated with the Chloroflexi and Proteobacteria phyla (Bienhold, Zinger, Boetius, & Ramette, 2016; Durbin & Teske, 2011, 2012; Russell, León‐Zayas, Wrighton, & Biddle, 2016; Walsh et al., 2015), as well as Marine Group II (MG‐II) archaea (Danovaro, Molari, Corinaldesi, & Dell'Anno, 2016). Nevertheless, chemoautotrophic organisms are often also present in oxic deep‐sea sediments, including the ammonia‐oxidizing Thaumarchaeota (Durbin & Teske, 2011; Lauer, Sørensen, & Teske, 2016). In addition, H_2_ produced by the radiolysis of water could serve as an energy source for H_2_‐oxidizing microorganisms in SPG sediments (Blair, D'Hondt, Spivack, & Kingsley, 2007; D'Hondt et al., 2015; Tully & Heidelberg, 2016). However, chemoautotrophic production in deep oxic sediments with depleted organic matter is limited by the availability of ammonia (Orsi, 2018; Wankel, Buchwald, Ziebis, Wenk, & Lehmann, 2015), and H_2_ (Bradley et al., 2018b; D'Hondt et al., 2015), and is expected to be a minor proportion of total microbial production at SPG. Our model focuses exclusively on aerobic heterotrophy, assuming that this is the dominant metabolism of the community within oxic SPG sediments, consistent with evidence from previous experimental and modelling studies (Bradley et al., 2018b; D'Hondt et al., 2009, 2015; LaRowe & Amend, 2015b). Genomic, metagenomic and expression‐based techniques offer promising means to better understand the taxonomic and metabolic diversity of marine sediment microbiota, as well as understanding dormancy regulation in subsurface environments. For example, metagenomic data could be explored to assess the distribution of orthologues of genes known or thought to be involved in dormancy in model taxa *Bacillus subtilis, Escherichia coli* and *Mycobacterium tuberculosis* (Asakura et al., 2006; Lennon & Jones, 2011; Sowell et al., 2008). Such genes include those which encode proteins involved in the regulation of entry into sporulation (Spo0A and Spo0B), toxin–antitoxin systems (including RelB–RelE, DinJ–YafQ, MazF–MazE and HipA–HipB) and resuscitation‐promoting factors (Rpfs) (Kana & Mizrahi, 2010; Lennon & Jones, 2011; Lewis, 2006; Piggot & Hilbert, 2004).

### 4.2 Model framework and parameter values

Parameter values are taken from the literature and constrained by measurements at SPG as well as calculations based on these data. No single value for maintenance powers (*m*_*B*1–4_) and death rates (*α*_*B*1–4_) can account for the observed biomass and POC concentrations over 75 million years of burial, thus justifying our selection of multiple pools representing biomass at different depths of dormancy (Lennon & Jones, 2011; Stolpovsky et al., 2011). Mortality rates (*α*_*Bn*_) are some of the most sensitive parameters. However, they are suitably constrained based on previous bioenergetic and non‐linear regression models (Bradley et al., 2018b), providing values from ~4 × 10^−6^ per year at the SWI to ~2 × 10^−8^ per year at 75 m sediment depth. Obtaining a measurement of microbial growth rates in marine sediments, on the other hand, has proved to be challenging, and the subject of whether microorganisms undergo growth at all in oligotrophic sediments is highly debated (Jørgensen & Marshall, 2016; Lever et al., 2015). A nominal value for *v*_*max*_ of 0.17 per thousand years is based on an estimate of biomass turnover time from ancient sediments on the Peruvian continental shelf (Lomstein et al., 2012), which is at the upper end of published estimations of turnover times from ancient marine sediments, spanning 1,000 to 73,000 years (Biddle et al., 2006; Jørgensen, 2011; Jorgensen et al., 2006; Lomstein et al., 2012; Whitman et al., 1998; Xie et al., 2013). Turnover times derived from amino acid racemization modelling are highly uncertain and have recently been revised (Braun et al., 2017). Despite a high degree of uncertainty associated with *v*_*max*_, cells at SPG are generally precluded from growth in any meaningful way due to the lack of available POC (POC << *K*_*v*_), and thus growth (*V*_*B*1_) is minimal. The half‐saturation constant for growth (*K*_*v*_) is a relatively poorly constrained parameter with a moderate influence on model results and must account not only for the concentration but also reactivity of POC, given the quasi‐1G approach implemented here. To derive more meaningful values for *v*_max_ and *K*_*v*_, future research should investigate the extent to which absolute (rather than net) microbial growth occurs in the marine subsurface, and how growth depends on substrate (and thus power) availability, POC reactivity and other factors.

In a natural environment, the bacterial growth yield, representing the proportion of organic carbon taken up that is incorporated into new biomass, is subject to trade‐offs based on a variety of selective pressures (Heijnen & Van Dijken, 1992; Lele & Watve, 2014; Sinsabaugh, Manzoni, Moorhead, & Richter, 2013). Distinguishing between true growth yield (*Y*_*G*_, Lipson, 2015) and maintenance activities is important in low or no‐growth environments, especially since maintenance activities potentially constitute a much greater fraction of total power utilization in these habitats (Bradley et al., 2018a; Kempes et al., 2017). A standalone maintenance requirement is used to mechanistically account for the utilization of large amounts of power by bacteria to serve functions that are not directly related to growth (Bradley et al., 2018a). The majority of estimates for bacterial growth efficiency for marine sediment microorganisms are within the range of 0.08 < *Y*_*G*_<0.20 (Biddle et al., 2006; D'Hondt, Wang, & Spivack, 2014; Heijnen & Van Dijken, 1992; Jørgensen & Marshall, 2016; Langerhuus et al., 2012; Lomstein et al., 2012; Starnawski et al., 2017; Whitman et al., 1998). We select the highest estimate (*Y*_*G *_= 0.20) since the published values typically do not distinguish growth yield from maintenance power requirement and thus are negatively skewed, accounting for the additional energy expended on maintenance activities (Jørgensen & Marshall, 2016; Whitman et al., 1998). Nevertheless, the model results are insensitive to variation in *Y*_*G*_ so the absolute value of *Y*_*G*_ is not important so long as maintenance activity is accounted for separately.

It is rare that studies simultaneously quantify respiration rates and cell numbers in deep marine sediments and thus estimates of per‐cell respiration (resulting from total growth and maintenance activities) are lacking (Hoehler & Jørgensen, 2013; Holmkvist et al., 2011; Leloup et al., 2007; Sahm, MacGregor, Jørgensen, & Stahl, 1999). We obtain estimates of maintenance power demand (*m*_*Bn*_) from a previous bioenergetic investigation of SPG sediments (Bradley et al., 2018b). A reactive continuum model was fit to measured POC data (D'Hondt et al., 2011) and is used to derive rates of POC degradation. These data are coupled to a non‐linear regression model for cell abundance, thus providing POC degradation rate per cell per depth (or equivalent sediment age). It is then assumed that cells are utilizing all energy derived from the oxidation of POC exclusively for maintenance (Bradley et al., 2018a). Based on these calculations, cells consume an amount of organic carbon equivalent to 2.0 to 2.9% of their C biomass per year for maintenance, and thus, a value of approximately 20–29 (per thousand years) can be attributed to *m*_*B*1_. Maintenance power demand declines in increasingly dormant cells, such that *m*_*B*1_ > *m*_*B*2_ > *m*_*B*3_ > *m*_*B*4_. Based on thermodynamic modelling and calculations provided in Bradley et al. (2018b), for *m*_*B*1* *_= 23 (per thousand years), maintenance power is equivalent to ~4 × 10^−19^ J s^−1^ cell^−1^, which is comparable to maintenance powers previously derived for SPG microbiota and the minimum power limit for microbial life (LaRowe & Amend, 2015b).

Parameters *R*_*S,D*_, *R*_*S,A*_, *K*_*S*_, *K*_*M*_, *st*_*S*_ and *st*_*M*_ are poorly constrained due to the arbitrary partitioning of microbial groups into discreet active and dormant pools. Therefore, it is challenging to determine meaningful values for these parameters based on experimentation, and accordingly, they are ideal for tuning and optimization exercises, and for maintaining model flexibility and transferability across timescales and/or habitats. Furthermore, there are other more sensitive parameters relating to growth, mortality and maintenance power demand that are not well defined that deserve greater attention in future work.

The model presented here should be considered a flexible framework upon which iterative improvements and adjustments can be made:

- Microbial diversity. Additional state variables could be added to account for a range of different metabolisms and functional groups.
- Physiology. The three states of dormancy resolved in the model (*B*_2–4_) are intended to represent a gradient of reduced metabolic activity rather than the existence of three distinct biological states, and additional or fewer physiological states may be included depending on the nature of the organisms and environment to be studied. It might also be useful to base microbial activity on thermodynamic factors or a measure of Gibbs energy, with thresholds and requirements based on an electron‐equivalent basis (e.g., Dale, Regnier, & Van Cappellen, 2006). Furthermore, the activation of dormant cells (i.e., transition from *B*_2–4_ to *B*_1_) and its mathematical formulation is not tested in the present setting, due to the continual worsening of conditions with increased burial at SPG. The nature of reactivation, as well as the rates associated with it, therefore, warrant further testing, which should draw from both laboratory and modelling studies (e.g., Morono et al., 2011; Stolpovsky, Fetzer, Van Cappellen, & Thullner, 2016; Stolpovsky et al., 2011; Takano et al., 2010; Trembath‐Reichert et al., 2017).
- Treatment of POC. We use a simple 1G approach to resolve organic carbon. 1G approaches have been widely applied to a range of sedimentary environments, are often favoured for sake of minimizing complexity and the number of parameters, and are suitable where the degradability of POC compounds does not vary widely (Arndt et al., 2013). However, unlike multi‐G and continuum approaches, a 1G model cannot capture the heterogeneity of sedimentary POC compounds and their various reactivity, as well as the various factors affecting POC degradation rate (Arndt et al., 2013). Nevertheless, the predictive capability of a more complex approach remains limited by the availability of appropriate measurements and data (e.g., molecular characterization of POC compounds, mineral–POC interactions, microbial functional groups), the level of mechanistic understanding of organic matter degradation rates in the environment and knowledge of appropriate parameter values. The 1G modelling approach implemented here is sufficient to provide a good fit between model results and measurements of cellular carbon and organic carbon concentration at SPG, while enabling us to focus our analyses on the novel components of the microbial model (i.e., bioenergetics and physiological state transitions). We suggest that the POC at SPG is likely to be comprised of a highly non‐reactive residual fraction of sedimentary organic matter (Bradley et al., 2018b), and thus may be appropriately lumped into a single pool (Arndt et al., 2013). Future iterations of this modelling framework might, however, expand on the treatment of POC to incorporate multiple or infinite pools.
- Transport. The 0D model can be easily expanded into higher dimensions (e.g., 1D) by including transport terms (Arndt et al., 2013).

## 5 CONCLUSIONS AND OUTLOOK

Microorganisms buried in SPG sediments undergo minimal to no new biomass growth; they transition to dormancy (with no reactivation over millions of years) and utilize substrate rather than biomass to meet their energetic demand for maintenance. By exploring dormancy and basal maintenance power requirements in a quantitative framework, it is clear that a microorganism's ability to transition between active and dormant physiological states, as well as the need to meet energetic maintenance power demand by exogenous strategies, are key factors in enabling its survival over long timescales. Thus, in the oligotrophic deep biosphere, the fitness of a microorganism may not be determined by its growth, but rather its ability simply to stay alive. Furthermore, the quantity of buried POC and the cost of maintenance activities set the ultimate limit on the duration that microorganisms can survive in a dormant state before endogenous catabolism results in rapid loss of cellular biomass. Recognizing the role of dormancy while quantifying the basal power requirement of organisms is critical to understanding how microorganisms endure unfavourable environments and to setting a limit on how long these organisms may survive in oligotrophic settings.
