# Supplementary material for: Survival of the fewest: Microbial dormancy and maintenance in marine sediments through deep time
Source: Geobiology. 2018 Sep 24;17(1):43–59. doi: 10.1111/gbi.12313 (PMC6585783; doi:10.1111/gbi.12313)
Supplement: Supplementary file 1 [file GBI-17-43-s001.pdf]

Supplementary information: Microbial dormancy and maintenance in marine sediments through deep time.

1. ReadMe: MicroLow 1.0

1.1 Hardware and Software Requirement

MicroLow 1.0 is written and can be executed in the free open source computing environment and programming language R, which is available for download on the web (http://www.r-project.org/). MicroLow 1.0 uses the adaptive time-step solver "lsoda" from the deSolve package (Soetaert et al., 2010) **which must also be installed**. On a standard desktop computer running R, the model usually takes less than 1 min to simulate 10 years of succession.

1.2 Download MicroLow 1.0

A package named "MICROLOW_1.0_source", containing the source code of MicroLow 1.0 and validation data is available at https://github.com/jbradley8365/MICROLOW_1.0_SOURCE.

The package contains:

The entire "MICROLOW_1.0_source" folder should be copied to a local computer such that the directory is: "/Users/jamesbradley/Documents/RFolder/MICROLOW_1.0_source/" **or** the folder paths contained in 'execute_MICROLOW_1.0.R' script should be modified (*setwd, path*, *pathte*) according to where the folder is copied to.

1.3 Description of files

This section provides a brief description of all files present in the folder "MICROLOW_1.0_source". This should be read alongside the following publication for clarity on variables, parameters, balance equations etc.:

Bradley, J. A., Amend, J. P., LaRowe, D. E., (2018) Microbial dormancy and maintenance in marine sediments through deep time. *Geobiology*.

MICROLOW_1.0_source contents:

ReadMe.docx / ReadMe.pdf
ReadMe guide to model source code and execution.

execute_MICROLOW_1.0.R
Model source code, .R script.

Biomass.dat
Validation date.
    Column 1: Sediment age (thousands of years)

Column 2: Cellular biomass carbon concentration (μg C cm^{-3}) at drill hole U1370, IODP Expedition 329, converted from cell abundance provided in D'Hondt et al. (2015) (see paper for details).

Corg.dat
Validation date.
Column 1: Sediment age (thousands of years)
Column 2: Particulate Organic Carbon (POC) concentration (μg C cm^{-3}) at drill hole U1370, IODP Expedition 329, converted from weight % measurements provided in D'Hondt et al. (2015) (see paper for details).

1.4 Model Operation

In R, specify working directory to appropriate path e.g.:
"Users/jamesbradley/Desktop/RFolder/MICROLOW_1.0_SOURCE/"

Open *execute_MICROLOW_1.0.R* script and execute in console (note: package "deSolve" (Soetaert et al., 2010) must be installed).

1.5 Output

Model output is created as variables within a dataframe 'out'. By default, output is not saved locally, however this can be done by using a command such as:

```
write.table(out_list[[1]],file=paste("out","nominal.csv",sep="_"),sep=",",row.names=FALSE)
```

The dataframe "out" contains the model results, with output provided for every thousand years simulated.

Variable	Description
time	Model run time, thousands of years
B1	B_1
B2	B_2
B3	B_3
B4	B_4
Corg	*POC*
c_Cons_Corg_Growth_B1	*POC* consumed by B_1 for growth, cumulative
c_Death_total	Death of B_{1-4}, cumulative
c_M_total_Corg	Exogenous maintenance of B_{1-4}, cumulative
c_M_total_bio	Endogenous maintenance of B_{1-4}, cumulative
c_M_total	Total maintenance of B_{1-4}, cumulative
c_M_B1_bio	Endogenous maintenance of B_1, cumulative
c_M_B1_Corg	Exogenous maintenance of B_1, cumulative
c_Cons_Corg_total	Total consumption of *POC*, cumulative
c_Growth_B1	Growth of B_1, cumulative
c_Death_B1	Death of B_1, cumulative
c_B1_D	Deactivation of B_1, cumulative
c_B2_A	Activation of B_2, cumulative
c_Death_B2	Death of B_2, cumulative
c_Death_B3	Death of B_3, cumulative
c_Death_B4	Death of B_4, cumulative
c_B2_D	Deactivation of B_2, cumulative
c_B3_D	Deactivation of B_3, cumulative

c_B3_A	Activation of B_3, cumulative
c_B4_A	Activation of B_4, cumulative
c_M_B2_bio	Endogenous maintenance of B_2, cumulative
c_M_B3_bio	Endogenous maintenance of B_3, cumulative
c_M_B4_bio	Endogenous maintenance of B_4, cumulative
years	Years simulated
rate_Cons_Corg_Growth_B1	Rate of *POC* consumed by B_1 for growth (year^{-1})
rate_Death_total	Rate of death of B_{1-4} (year^{-1})
rate_M_total_Corg	Rate of exogenous maintenance of B_{1-4} (year^{-1})
rate_M_total_bio	Rate of endogenous maintenance of B_{1-4} (year^{-1})
rate_M_total	Rate of maintenance of B_{1-4} (year^{-1})
rate_M_B1_bio	Rate of endogenous maintenance of B_1 (year^{-1})
rate_M_B1_Corg	Rate of exogenous maintenance of B_1 (year^{-1})
rate_Cons_Corg_total	Rate of consumption of *POC* (year^{-1})
rate_Growth_B1	Rate of growth of B_1 (year^{-1})
rate_Death_B1	Rate of death of B_1 (year^{-1})
rate_B1_D	Rate of deactivation of B_1 (year^{-1})
rate_B2_A	Rate of activation of B_2 (year^{-1})
rate_Death_B2	Rate of death of B_2 (year^{-1})
rate_Death_B3	Rate of death of B_3 (year^{-1})
rate_Death_B4	Rate of death of B_4 (year^{-1})
rate_B2_D	Rate of deactivation of B_2 (year^{-1})
rate_B3_D	Rate of deactivation of B_3 (year^{-1})
rate_B3_A	Rate of activation of B_3 (year^{-1})
rate_B4_A	Rate of activation of B_4 (year^{-1})
rate_M_B2_bio	Rate of endogenous maintenance of B_2 (year^{-1})
rate_M_B3_bio	Rate of endogenous maintenance of B_3 (year^{-1})
rate_M_B4_bio	Rate of endogenous maintenance of B_4 (year^{-1})
Theta_S	θ_S
Theta_M	θ_M
Btotal	Total biomass (B_{1-4})

References

Soetaert, K., Petzoldt, T., and Setzer, R. W. (2010). Solving Differential Equations in R: Package deSolve. *J. Stat. Softw.* 33, 1–25.

2. Executable code

```
## MicroLow 1.0

# James Bradley
# jbradley8365@gmail.com
# University of Southern California


# PUBLICATION:
# Bradley, J. A., Amend, J. P., LaRowe, D. E., (2018)
# Microbial dormancy and maintenance in marine sediments through deep time.
# Geobiology.


# EXECUTE THIS CODE TO RUN MODEL


# 1.0 SET UP WORKSPACE

# Clear workspace

rm(list = ls())
```

```
# Set working directory

setwd("/Users/jamesbradley/Desktop/RFolder/MICROLOW_1.0_SOURCE/")

# Download, install and run Packages


library("rootSolve", lib="/Users/jamesbradley/Desktop/RFolder/New_Packages")
library("shape", lib="/Users/jamesbradley/Desktop/RFolder/New_Packages")
library("deSolve", lib="/Users/jamesbradley/Desktop/RFolder/New_Packages")
library("ReacTran", lib="/Users/jamesbradley/Desktop/RFolder/New_Packages")
library("bvpSolve", lib="/Users/jamesbradley/Desktop/RFolder/New_Packages")
library("scatterplot3d", lib="/Users/jamesbradley/Desktop/RFolder/New_Packages")


######
######

# 2.0 SPECIFY PATHS

path    <-"/Users/jamesbradley/Desktop/RFolder/MICROLOW_1.0_SOURCE/"
pathte  <-"/Users/jamesbradley/Desktop/RFolder/MICROLOW_1.0_SOURCE/"


######
######


# 3.0 LOAD VALIDATION DATA

# Data from D'Hondt et al (2015) Nature Geoscience, IODP 329, South Pacific Gyre, U1370

biomass_obs<-read.table(paste(pathte,"Biomass.dat",sep=""),header=FALSE)
time_biomass<-biomass_obs[,1]*1000
value_biomass<-biomass_obs[,2]

Corg_obs<-read.table(paste(pathte,"Corg.dat",sep=""),header=FALSE)
time_Corg<-Corg_obs[,1]*1000
value_Corg<-Corg_obs[,2]

######
######

# 4.0 DEFINE TIMEPERIOD

# nyears is in THOUSANDS OF YEARS
# for 1000 years, nyears = 1

nyears<-80000

times<-seq(0,nyears,by=1)      # Times at which model provides output

######
######


# 5.0 SET PARAMETER VALUES

V_max_B1 <- 0.173286        # Maximum growth rate of B1
K_V_B1 <- 40000             # Half-saturation constant for growth
YG <- 0.2                   # True growth yield
mq_B1 <- 23                 # Maintenance demand B1
mq_B2 <- 19                 # Maintenance demand B2
mq_B3 <- 15                 # Maintenance demand B3
mq_B4 <- 11                 # Maintenacne demand B4
alpha_B1 <- 0.003500        # Mortality rate B1
alpha_B2 <- 0.00082         # Mortality rate B2
alpha_B3 <- 0.00011         # Mortality rate B3
alpha_B4 <- 0.000014        # Mortality rate B4
st_S <- 0.1                 # Steepness of state-change function
K_Corg_S <- 10000           # Threshold POC for state-change
R_S_D  <- 0.0001            # Rate constant, deactivation
R_S_A  <- R_S_D             # Rate constant, activation
st_M <- 0.1                 # Steepness of maintenance function
K_Corg_M <- 15              # Threshold POC for maintenance energy provenance



parms<-c(
  V_max_B1,
  K_V_B1,
  YG,
  mq_B1,
  mq_B2,
  mq_B3,
  mq_B4,
  alpha_B1,
```

```
  alpha_B2,
  alpha_B3,
  alpha_B4,
  st_S,
  K_Corg_S,
  R_S_D,
  R_S_A,
  st_M,
  K_Corg_M
)


######
######

# 6.0 LOAD DRIVERS

#none


# 7.0 INITIAL VALUES

start<-c(B1=0.040,
         B2=0.0,
         B3=0.0,
         B4=0.0,
         Corg=781.259,
         c_Cons_Corg_Growth_B1 = 0,
         c_Death_total = 0,
         c_M_total_Corg = 0,
         c_M_total_bio = 0,
         c_M_total = 0,
         c_M_B1_bio = 0,
         c_M_B1_Corg = 0,
         c_Cons_Corg_total = 0,
         c_Growth_B1 = 0,
         c_Death_B1 = 0,
         c_B1_D = 0,
         c_B2_A = 0,
         c_Death_B2=0,
         c_Death_B3=0,
         c_Death_B4=0,
         c_B2_D=0,
         c_B3_D=0,
         c_B3_A=0,
         c_B4_A=0,
         c_M_B2_bio=0,
         c_M_B3_bio=0,
         c_M_B4_bio=0
)

#...................................................................................


# 8.0 CONSTRUCT ARRAYS FOR OUTPUT

out_list = list()


# 9.0 MODEL DEFINITION AND EXECUTION WITHIN COUNTER

for(counter in 1:1) {

  # 9.1 BEGINNING OF MODEL DEFINITION

  model<-function(t,xx,parms){

    B1<-xx[1]
    B2<-xx[2]
    B3<-xx[3]
    B4<-xx[4]
    Corg<-xx[5]
    c_Cons_Corg_Growth_B1<-xx[6]
    c_Death_total<-xx[7]
    c_M_total_Corg<-xx[8]
    c_M_total_bio<-xx[9]
    c_M_total<-xx[10]
    c_M_B1_bio<-xx[11]
    c_M_B1_Corg<-xx[12]
    c_Cons_Corg_total <-xx[13]
    c_Growth_B1<-xx[14]
    c_Death_B1<-xx[15]
    B1_D<-xx[16]
    B2_A<-xx[17]
```

```
Death_B2<-xx[18]
Death_B3<-xx[19]
Death_B4<-xx[20]
B2_D<-xx[21]
B3_D<-xx[22]
B3_A<-xx[23]
B4_A<-xx[24]
M_B2_bio<-xx[25]
M_B3_bio<-xx[26]
M_B4_bio<-xx[27]

with(as.list(parms),{

  # Switch function for state change
  Theta_S <- 1/(exp((-Corg+K_Corg_S)/(st_S*K_Corg_S))+1)

  #Switch function for provenance of maintenance energy
  Theta_M <- 1/(exp((-Corg+K_Corg_M)/(st_M*K_Corg_M))+1)

  # Growth
  Growth_B1 <- V_max_B1*B1*(Corg/(Corg+K_V_B1))

  # Death
  Death_B1 <- alpha_B1*B1
  Death_B2 <- alpha_B2*B2
  Death_B3 <- alpha_B3*B3
  Death_B4 <- alpha_B4*B4

  Death_total = Death_B1 + Death_B2 + Death_B3 + Death_B4

  # Activation and deactivation

  B2_A <- Theta_S*R_S_A*B2
  B1_D <- (1-Theta_S)*R_S_D*B1
  B3_A <- Theta_S*R_S_A*B3
  B2_D <- (1-Theta_S)*R_S_D*B2
  B4_A <- Theta_S*R_S_A*B4
  B3_D <- (1-Theta_S)*R_S_D*B3

  # Consumption of substrate due to active biomass growth

  Cons_Corg_Growth_B1 <- Growth_B1*(1/YG)


  # Endogenous maintenance

  M_B1_bio = mq_B1*B1*(1-Theta_M)
  M_B2_bio = mq_B2*B2*(1-Theta_M)
  M_B3_bio = mq_B3*B3*(1-Theta_M)
  M_B4_bio = mq_B4*B4*(1-Theta_M)

  M_total_bio = M_B1_bio + M_B2_bio + M_B3_bio + M_B4_bio

  # Exogenous maintenance

  M_B1_Corg = mq_B1*B1*Theta_M
  M_B2_Corg = mq_B2*B2*Theta_M
  M_B3_Corg = mq_B3*B3*Theta_M
  M_B4_Corg = mq_B4*B4*Theta_M

  M_total_Corg = M_B1_Corg + M_B2_Corg + M_B3_Corg + M_B4_Corg

  # Total maintenance (from biomass and from organic carbon)

  M_total = M_total_bio + M_total_Corg

  # Total substrate consumption

  Cons_Corg_total = Cons_Corg_Growth_B1 + M_total_Corg


  # BALANCE EQUATIONS

  dB1 <-  Growth_B1 - B1_D - Death_B1 - M_B1_bio + B2_A + B3_A +  B4_A

  dB2 <-  B1_D - Death_B2 - B2_A - M_B2_bio - B2_D

  dB3 <-  B2_D - Death_B3 - B3_A - M_B3_bio - B3_D

  dB4 <-  B3_D - Death_B4 - B4_A - M_B4_bio

  dCorg <- Death_total - Cons_Corg_total


  #Derived variables

  dc_Cons_Corg_Growth_B1 <- Cons_Corg_Growth_B1
```

```
      dc_Death_total <- Death_total

      dc_M_total_Corg <- M_total_Corg

      dc_M_total_bio <-   M_total_bio

      dc_M_total <- M_total

      dc_M_B1_bio <- M_B1_bio

      dc_M_B1_Corg <- M_B1_Corg

      dc_Cons_Corg_total <- Cons_Corg_total

      dc_Growth_B1 <- Growth_B1
      dc_Death_B1 <- Death_B1
      dc_B1_D <- B1_D
      dc_B2_A <- B2_A

      dc_Death_B2<- Death_B2
      dc_Death_B3<- Death_B3
      dc_Death_B4<- Death_B4
      dc_B2_D<- B2_D
      dc_B3_D<- B3_D
      dc_B3_A<- B3_A
      dc_B4_A<- B4_A

      dc_M_B2_bio<-M_B2_bio
      dc_M_B3_bio<-M_B3_bio
      dc_M_B4_bio<-M_B4_bio

      # List the state variables and derived variables for which you want output

      list(c(dB1, dB2, dB3, dB4, dCorg, dc_Cons_Corg_Growth_B1, dc_Death_total,
dc_M_total_Corg, dc_M_total_bio, dc_M_total, dc_M_B1_bio,
dc_M_B1_Corg,dc_Cons_Corg_total,dc_Growth_B1, dc_Death_B1, dc_B1_D,
dc_B2_A,dc_Death_B2,dc_Death_B3, dc_Death_B4, dc_B2_D, dc_B3_D, dc_B3_A, dc_B4_A,
dc_M_B2_bio,dc_M_B3_bio,dc_M_B4_bio))

    })
  }


  # 9.2 END OF MODEL DEFINITION (STILL WITHIN LOOP)

  out<-as.data.frame(lsoda(start,times,model,parms))

  # Derived variables and rates

  out$years<-out$time*1000

  out$rate_Cons_Corg_Growth_B1[2:(nrow(out))]<-(out$c_Cons_Corg_Growth_B1[2:(nrow(out))] -
out$c_Cons_Corg_Growth_B1[1:(nrow(out)-1)])/1000
  out$rate_Cons_Corg_Growth_B1[1]=0

  out$rate_Death_total[2:(nrow(out))]<-(out$c_Death_total[2:(nrow(out))] -
out$c_Death_total[1:(nrow(out)-1)])/1000
  out$rate_Death_total[1]=0

  out$rate_M_total_Corg[2:(nrow(out))]<-(out$c_M_total_Corg[2:(nrow(out))] -
out$c_M_total_Corg[1:(nrow(out)-1)])/1000
  out$rate_M_total_Corg[1]=0

  out$rate_M_total_bio[2:(nrow(out))]<-(out$c_M_total_bio[2:(nrow(out))] -
out$c_M_total_bio[1:(nrow(out)-1)])/1000
  out$rate_M_total_bio[1]=0

  out$rate_M_total[2:(nrow(out))]<-(out$c_M_total[2:(nrow(out))] -
out$c_M_total[1:(nrow(out)-1)])/1000
  out$rate_M_total[1]=0

  out$rate_M_B1_bio[2:(nrow(out))]<-(out$c_M_B1_bio[2:(nrow(out))] -
out$c_M_B1_bio[1:(nrow(out)-1)])/1000
  out$rate_M_B1_bio[1]=0

  out$rate_M_B1_Corg[2:(nrow(out))]<-(out$c_M_B1_Corg[2:(nrow(out))] -
out$c_M_B1_Corg[1:(nrow(out)-1)])/1000
  out$rate_M_B1_Corg[1]=0

  out$rate_Cons_Corg_total[2:(nrow(out))]<-(out$c_Cons_Corg_total[2:(nrow(out))] -
out$c_Cons_Corg_total[1:(nrow(out)-1)])/1000
  out$rate_Cons_Corg_total[1]=0

  out$rate_Growth_B1[2:(nrow(out))]<-(out$c_Growth_B1[2:(nrow(out))] -
out$c_Growth_B1[1:(nrow(out)-1)])/1000
  out$rate_Growth_B1[1]=0
```

```
  out$rate_Death_B1[2:(nrow(out))]<-(out$c_Death_B1[2:(nrow(out))] -
out$c_Death_B1[1:(nrow(out)-1)])/1000
  out$rate_Death_B1[1]=0

  out$rate_B1_D[2:(nrow(out))]<-(out$c_B1_D[2:(nrow(out))] - out$c_B1_D[1:(nrow(out)-
1)])/1000
  out$rate_B1_D[1]=0

  out$rate_B2_A[2:(nrow(out))]<-(out$c_B2_A[2:(nrow(out))] - out$c_B2_A[1:(nrow(out)-
1)])/1000
  out$rate_B2_A[1]=0

  out$rate_Death_B2[2:(nrow(out))]<-(out$c_Death_B2[2:(nrow(out))] -
out$c_Death_B2[1:(nrow(out)-1)])/1000
  out$rate_Death_B2[1]=0

  out$rate_Death_B3[2:(nrow(out))]<-(out$c_Death_B3[2:(nrow(out))] -
out$c_Death_B3[1:(nrow(out)-1)])/1000
  out$rate_Death_B3[1]=0

  out$rate_Death_B4[2:(nrow(out))]<-(out$c_Death_B4[2:(nrow(out))] -
out$c_Death_B4[1:(nrow(out)-1)])/1000
  out$rate_Death_B4[1]=0

  out$rate_B2_D[2:(nrow(out))]<-(out$c_B2_D[2:(nrow(out))] - out$c_B2_D[1:(nrow(out)-
1)])/1000
  out$rate_B2_D[1]=0

  out$rate_B3_D[2:(nrow(out))]<-(out$c_B3_D[2:(nrow(out))] - out$c_B3_D[1:(nrow(out)-
1)])/1000
  out$rate_B3_D[1]=0

  out$rate_B3_A[2:(nrow(out))]<-(out$c_B3_A[2:(nrow(out))] - out$c_B3_A[1:(nrow(out)-
1)])/1000
  out$rate_B3_A[1]=0

  out$rate_B4_A[2:(nrow(out))]<-(out$c_B4_A[2:(nrow(out))] - out$c_B4_A[1:(nrow(out)-
1)])/1000
  out$rate_B4_A[1]=0

  out$rate_M_B2_bio[2:(nrow(out))]<-(out$c_M_B2_bio[2:(nrow(out))] -
out$c_M_B2_bio[1:(nrow(out)-1)])/1000
  out$rate_M_B2_bio[1]=0

  out$rate_M_B3_bio[2:(nrow(out))]<-(out$c_M_B3_bio[2:(nrow(out))] -
out$c_M_B3_bio[1:(nrow(out)-1)])/1000
  out$rate_M_B3_bio[1]=0

  out$rate_M_B4_bio[2:(nrow(out))]<-(out$c_M_B4_bio[2:(nrow(out))] -
out$c_M_B4_bio[1:(nrow(out)-1)])/1000
  out$rate_M_B2_bio[1]=0




  # COMPUTE TOTALS AND ASSIGN TO NEW VARIABLES

  out$Theta_S <- 1/(exp((-out$Corg+K_Corg_S)/(st_S*K_Corg_S))+1)

  out$Theta_M <- 1/(exp((-out$Corg+K_Corg_M)/(st_M*K_Corg_M))+1)

  out$Btotal<- out$B1 + out$B2 + out$B3  + out$B4



  out_list[[counter]]<-out

}

# END OF MODEL RUN

#####
#####

# 10.0 PLOTTING AND RESULTS

par(mfrow = c(3, 2))
plot(out_list[[1]]$years,out_list[[1]]$B1,type='l', xlab='years', main='B1 biomass',
ylab='ugC/cm3')
plot(out_list[[1]]$years,out_list[[1]]$B2,type='l', xlab='years', main='B2 biomass',
ylab='ugC/cm3')
plot(out_list[[1]]$years,out_list[[1]]$B3,type='l', xlab='years', main='B3 biomass',
ylab='ugC/cm3')
plot(out_list[[1]]$years,out_list[[1]]$B4,type='l', xlab='years', main='B4 biomass',
ylab='ugC/cm3')
```

```
plot(out_list[[1]]$years,out_list[[1]]$Btotal,type='l', xlab='years',main='Total biomass',
ylab='ugC/cm3', log="y", ylim=c(0.000004,0.04))
points(time_biomass,value_biomass, col="red2")

plot(out_list[[1]]$years,out_list[[1]]$Corg,type='l', xlab='years', main='Organic C',
ylab='ugC/cm3',ylim=c(0,800))
points(time_Corg,value_Corg, col="red2")
```
